# Cold plasma treatment to release dormancy and improve growth in grape buds: a promising alternative to natural chilling and rest breaking chemicals

**DOI:** 10.1038/s41598-020-59097-x

**Published:** 2020-02-14

**Authors:** Z. Mujahid, T. Tounekti, H. Khemira

**Affiliations:** 1 Department of Physics, Faculty of Science, Jazan University, Jazan, 45142 Saudi Arabia; 2 Centre for Environmental Research & Studies, Jazan University, Jazan, 45142 Saudi Arabia

**Keywords:** Enzymes, Plant biotechnology, Plasma physics

## Abstract

Winter dormancy of temperate zone perennial plant species is commonly released by chilling temperature. If the duration of the cold weather is not adequate, plant growth becomes disorganized leading to reduced growth, spread out flowering and fruit maturation and often reduced yield. In mild-winter regions, growers commonly resort to spraying their trees with chemicals such as hydrogen cyanamide to compensate for the lack of chilling to ensure good growth and yield. Although effective, most of these chemicals are highly toxic; unfortunately, there is no effective and environmentally friendly alternative which can be used to release dormancy. In this work, we present a cold plasma treatment-based method which can effectively release the dormancy of grape buds. We have found that exposing grape buds to plasma provides improvement of several growth parameters including higher, faster and more synchronous budbreak and more vigorous vegetative growth, comparatively similar to or better than natural chilling. Biochemical analyses of bud tissue suggest that the plasma treatment triggered a marked transient oxidative stress as indicated by the increase in the concentrations of free proline, malondialdehyde (MDA) and hydrogen peroxide (H_2_O_2_). Proline appears to have played a key role; as a compatible osmolyte, it may have protected cellular structures against free radicals and as a signaling molecule, it may have induced the events leading to dormancy release. We anticipate that our work will provide a starting point for the development of novel plasma-based tools and methods to treat dormant plants. The plasma treatment method may allow higher agricultural production in several regions of the world at risk of becoming marginal for the cultivation of certain crops due to global warming.

## Introduction

Several fruit vines such as grape and kiwi, fruit and nut trees and ornamental woody species cease active growth in early fall, lose their leaves then become dormant in the winter. Such plants are considered as endodormant, i.e., their growth is inhibited from within the dormant structures themselves (the buds)^1^. Under natural conditions, endodormancy is released by the cold of winter. Once the chilling requirement of the plant’s structure (bud or seed) is met, it resumes active growth if environmental conditions are suitable^2,3^. If the chilling requirement of a plant is not met such as in mild-winter regions, budbreak becomes erratic and asynchronous leading to reduced vegetative growth, spread out flowering and fruit maturation and often reduced yield^4–6^. Increasingly, temperate zone fruit trees and vines are cultivated in warmer regions beyond their natural habitat as well as temperatures are rising due to global warming and climatic change, which makes dormancy critical for future agriculture^7^.

Currently, growers resort to the application of artificial rest-breaking agents such as thiourea, calcium cyanamide, potassium nitrate and hydrogen cyanamide to promote budbreak and manipulate flowering of the trees^2^. For instance, hydrogen cyanamide-based chemicals have been used effectively on several fruit crops such grapevines^8–10^ and kiwi vines^11^. However, the effectiveness of the treatment depends on the time of application^9^; besides, the use of this chemical has been restricted in several countries because of its high toxicity to humans and wildlife. Furthermore, most of these chemicals are phytotoxic to the plant itself and neighbouring crops. Therefore, there is an urgent need to find safer but equally effective alternatives to hydrogen cyanamide.

Rest-breaking chemicals such as hydrogen cyanamide were shown to cause sub-lethal stress within the treated tissue which leads to endodormancy release^12^; the mechanisms involved are still not well understood. The available evidence indicates that dormancy interruption is tightly associated with oxidative processes taking place in the dormant structure^13–15^. In dormant grapevine buds, hydrogen cyanamide inhibits both the activity of the antioxidant enzyme catalase (CAT, EC 1.11.1.6) and the expression of its gene^13^ leading to a transient accumulation of hydrogen peroxide (H_2_O_2_) which regulates the release of endodormancy and budbreak^15,16^. Most genes expressed during dormancy release are related to the oxidative processes and stress responses within the cell^17^. It was therefore suggested that grape dormancy and its release are controlled by the changes in H_2_O_2_ metabolism. However, how hydrogen cyanamide exactly affects the reactive oxygen species (ROS) regulatory network during grapevine dormancy release remains unclear. The hydrogen cyanamide treatment also stimulates the accumulation of the amino acid proline and the polyamine putrescine which act as free radical scavengers, molecular messengers and also as sources of nitrogen and carbon for the rapid tissue growth which follows budbreak^15,18^.

Cold atmospheric plasma has recently emerged as a promising simple, low-cost and efficient tool for the disinfection of water and food and even human skin^19–21^. In addition to the sterilisation of articles and surfaces, it is investigated worldwide for the treatment of plant seeds^22–26^. Plasma can generate electron, ions, radicals, reactive species and light radiation including UV^27^. Part or a combination of these components have been utilised for the treatment of the seeds to induce biological responses such as germination^26,28,29^, seedling growth^22,24,30^, stress tolerance^31^ and improvement in yield^32^. They have been tested on seeds of several species such as tomato^33^, wheat^28^, sunflower^22^ and quinoa^23^. It was observed that the plasma etches the external surface of the seed teguments^34^; this possibly facilitates imbibition and advances germination.

In the present work, we report a novel effect of plasma treatment of dormant grape shoots which results in the release of bud dormancy. This study examined the effect of plasma treatment on growth and antioxidants’ status in dormant buds of *Vitis vinefera* L. cv. Muscat of Alexandria. We evaluated the changes in CAT activity and the concentrations of H_2_O_2_, Malondialdehyde (MDA) which is considered a marker of damage due to oxidative stress and proline. Our results show that plasma-based treatment can advance and synchronize budbreak and increase shoot vigour. The literature research suggests that this is the first report on the use of plasma to break bud dormancy in any plant species.

## Materials and Methods

### Plant material

Initial experiments were carried out in small replicates on shoots of the local grape cultivar El-Reizki, for widespread conditions of gas, treatment time, power and plasma type. Some of the plasma treatments stood out to be very effective in releasing bud dormancy.

More detailed experiments were performed on lignified canes of the common grape cultivar Muscat of Alexandria with a diameter of 0.4 to 1 cm which were collected in late November from several vines in a vineyard in Abha mountains region of Saudi Arabia. The canes were cut into short single-node segments to preclude the influence of apical dominance and to allow the material to fit into the plasma chamber.

In total, five distinct sample were studied. Three sets of cuttings were treated with plasma for 2, 5 or 10 minutes. The fourth set of cuttings were wrapped in a plastic bag and kept in a refrigerator at 4 °C for one month so to receive enough chilling to release their dormancy (the equivalent of 720 chill units). The fifth set of cuttings was left untreated to serve as a control.

For the monitoring of growth, each condition was replicated four times, and each replicate consisted of 20-single-node cuttings. After the treatments, the basal ends of the shoots were cut under water to prevent air bubbles from forming inside the xylem vessels which would inhibit water uptake^35^. The cuttings were then placed in trays containing distilled water such that ~1cm of their base remained immersed. The trays were placed in a growth chamber set to 22 °C, 14 H light- and 18 °C, 10 H dark-period to force the buds to grow. Relative humidity was maintained at 95% and fluorescence light having a flux density of 150 μmol m^−2^ s^−1^ was used. The phenological stage of each bud was assessed every two days for 16 days. The buds were assigned with standard growth stages, i.e. dormant, swollen, green tip (when green tissue becomes visible) and open (when at least one fully expanded leaf becomes visible).

At the end of the forcing period, the largest leaf from each cutting (which grew) was harvested and its area was determined with a leaf Area Meter (LI-3100C). The average area per leaf was then calculated for each replicate. The percentage of cuttings which rooted was also determined for each treatment.

To obtain bud tissue for biochemical analyses, each of the treatments control, 5-min plasma and chilling were applied to extra three sets of 30-cutting each as descried previously. These cuttings were then forced under the same conditions as the previous ones. Samples of buds were taken from these cuttings at 2, 8 and 14 days after treatment (DAT). These samples were immediately frozen in liquid nitrogen then stored at −50 °C for future biochemical analyses.

### Plasma generation apparatus and treatment conditions

The schematic of the planar Dielectric Barrier Discharge (DBD) used in this work is included as supplementary material (Supplementary Figure S1). The planar DBD consists of a 10 cm internal diameter, 1 cm height quartz ring with two pipe connectors on the side for the gas inlet and outlet. The top and bottom are covered by 1-mm thick glass plates. The two electrodes are present outside above and below the glass plates. The top glass plate can be removed to insert the samples to be treated. Two mass flow controllers were used to provide a constant flow of 2 litres / min of Helium gas (99.99% purity) with 0.5% admixture of oxygen which enter the chamber from one side through a tube and exit from the other. For plasma treatment, the buds were placed inside an air-tight quartz chamber without any metal parts. They were treated for specified time duration, at a constant applied power of 30 W. The plasma treatments lasted 2, 5 or 10 minutes..

### Biochemical analyses

#### Catalase activity

For the biochemical analyses, bud tissues were grinded in liquid nitrogen, then 100 mg of powdered tissue was extracted with 1 mL of cold potassium phosphate buffer (50 mM, pH 7.5) containing 2% (w/v) PVP and EDTA (1 mM)^15^. After centrifugation at 4 °C, the supernatants were directly used for measuring protein content and assaying enzyme activity. The activity of CAT was measured spectrophotometrically using the change in absorbance at 240 nm of a solution containing H_2_O_2_ using an extinction coefficient of 39.3 mM^−1^ cm^−1^. The change in absorbance at per minute and milligram of protein gives us specific activity of the enzyme. Total soluble protein concentration is determined according to Bradford^36^, using BSA as a standard.

#### Proline content

Proline concentration in the bud extract was determined by the ninhydrin method as described by Bates *et al.*^37^. In short, 200 mg of powdered dry bud tissue was extracted for 30 min with 5 mL of boiling methanol (40%, v/v). Two millilitres of glacial acetic acid and 1 mL of the ninhydrin solution (consisting of 25% distilled water, 60% glacial acetic acid and 15% orthophosphoric acid) were added to 1 mL of the methanolic extract. The mixture was boiled for 30 min, and toluene (3 ml) was added after cooling the mixture on ice. The upper phase was separated then dehydrated with NaSO_4_. After incubation of the solution in the dark for at least two hours, the absorbance was read at 528 nm.

#### Malondialdehyde concentration

Malondialdehyde levels were determined using the method described by^38^, which corrects for the interference of impurities in the thiobarbituric acid (TBA) reactive substances assay. Briefly, buds were repeatedly extracted with 80:20 (v/v) ethanol/water containing 1 mg mL^−1^ butylated hydroxytoluene (BHT) using ultra-sonication. This was followed by centrifugation; then the supernatants were pooled and an aliquot of sample was combined with a volume of either (1) −TBA solution containing 20% (w/v) trichloroacetic acid and 0.01% (w/v) BHT, or (2) + TBA solution containing the above plus 0.65% (w/v) TBA. Those samples were incubated in test tubes at 95°C for 25 min and, after cooling, absorbance was read at 440, 532, and 600 nm. The following formula was used to calculate MDA equivalents (nmol mL^−1^):

$$106\times (({\rm{A}}-{\rm{B}})/157,000),$$

where A = ((Abs 532 + TBA) − (Abs 600 + TBA) − (Abs 532 − TBA − Abs 600 − TBA)), and B = ((Abs 440 + TBA − Abs 600 + TBA) × 0.0571).

#### Hydrogen peroxide (H_2_O_2_) content

The concentration of H_2_O_2_ (nmol g^−1^ fresh weight) in bud tissue was determined as described by^39^. In short, bud samples (about 300 mg) were ground into a fine powder in liquid nitrogen then homogenized in 3 ml of 1% (w/v) trichloroacetic acid (TCA). The extracts were centrifuged at 10,000 × g (4 °C) for 10 min. The supernatant was collected, filtered and 0.75 ml of the supernatants was added to 0.75 ml of 10 mM Potassium phosphate buffer (pH 7.0) and 1.5 ml of 1 M potassium iodide (KI). H_2_O_2_ content was determined at 390 nm using a standard calibration curve.

### Statistical analyses

The data were subjected to analysis of variance (ANOVA) using OriginPro 2018 software. A Completely Randomized Design with four replicates of 20 cuttings each was used for growth analysis. Where applicable, the means were separated by Tukey test with a level of significance *P* = 0.05. A similar analysis procedure was used for biochemical analyses results but only three replicates of 30 cuttings each were used.

## Results

**Figure 1:**
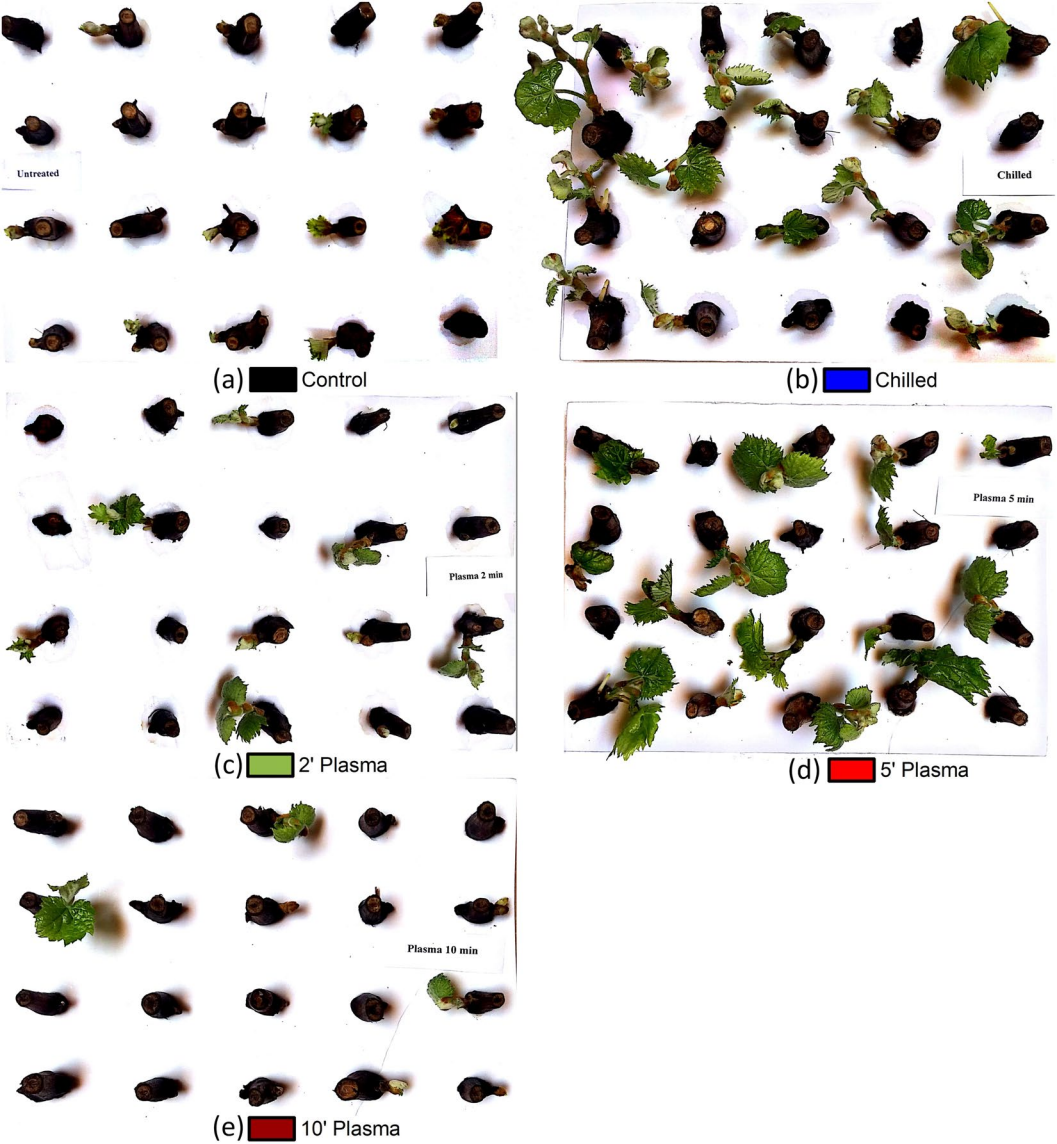
Growth of buds on single-node cuttings of Muscat of Alexandria grape after 10 days of forcing under 22 °C, 14 H light- and 18 °C, 10 H dark-period conditions. Prior to forcing, the cuttings were either untreated (control) (**a**), chilled for 1-month at 4 °C (**b**) or treated with cold atmospheric plasma for 2 (**c**), 5 (**d**) or 10 (**e**) min. Each picture represents one out of the four replicates of each treatment.

**Figure 2:**
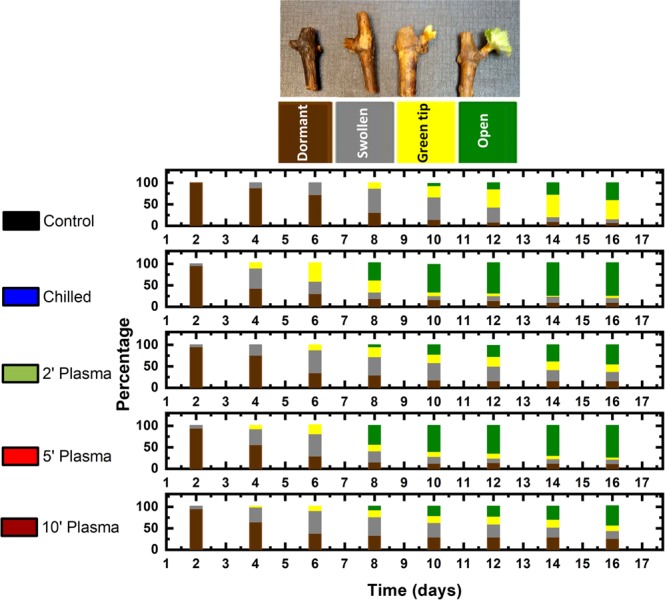
The percentage of buds at each phenological stage as a function of days of forcing under 22 °C, 14 H light- and 18 °C, 10 H dark-period conditions on Muscat of Alexandria grape shoot cuttings. Prior to forcing, the cuttings were either untreated (control), chilled for 1-month at 4 °C or treated with cold atmospheric plasma for 2, 5 or 10 min. Each bar represents the mean of four replicates of 20 cuttings each. A picture of specimens of buds representing each of the four phenological stages of dormant, swollen, green-tip and budburst is shown above the histogram.

The pictures of untreated control, chilled, plasma-treated shoots of the grape variety Muscat of Alexandria, after ten days of forcing are shown in Fig. 1. In comparison to the untreated control buds (a), both the chilled (b) and 5-mn plasma-treated shoots (d) had an earlier budbreak and a higher percentage of open buds (as shown quantitatively in Fig. 2). The 2-min (c) and 10-min (e) treatments did also have slightly earlier bud development in comparison to control; however, the final percentage of open buds after 16 days of forcing was comparable at 39–44%. The 50% budbreak mark was reached on the 9^th^ day of forcing by chilled and 5 minutes plasma-treated cuttings, that’s a 9-day advance in budbreak compared to control shoots.

The distribution of buds among four early phenological stages every two days during forcing is depicted as stacked bar histograms in Fig. 2. The picture of specimens of buds at each of the four phenological stages of dormant, swollen, green-tip and budburst is shown above the histogram and marked with their representative colour as in the bar chart. The dormant (brown) refers to the state when the bud has no sign of growth. The swollen state (grey) refers to the slight increase in the size of the bud and opening of the scales. The green tip (yellow) signifies the stage when some green tissue was visible, but the leaves did not unfold yet. Budburst is the final stage when at least one leaf has fully emerged from the bud.

The control group in Fig. (2) indicates buds which were forced without any prior treatment. These buds did not show any sign of activity during the first seven days of forcing and the first signs of swelling appeared after nine days. After 16 days, about 50% of the buds reached green tip and about 40% reached budburst stages. The buds chilled in 4 °C cold for one month started to swell just after four days of forcing, much earlier than the untreated control buds. After 14 days of forcing, about 75% of the buds were open. The growth pattern of the cuttings which were exposed to plasma for 5 minutes was similar to that of the chilled cuttings.

**Figure 3:**
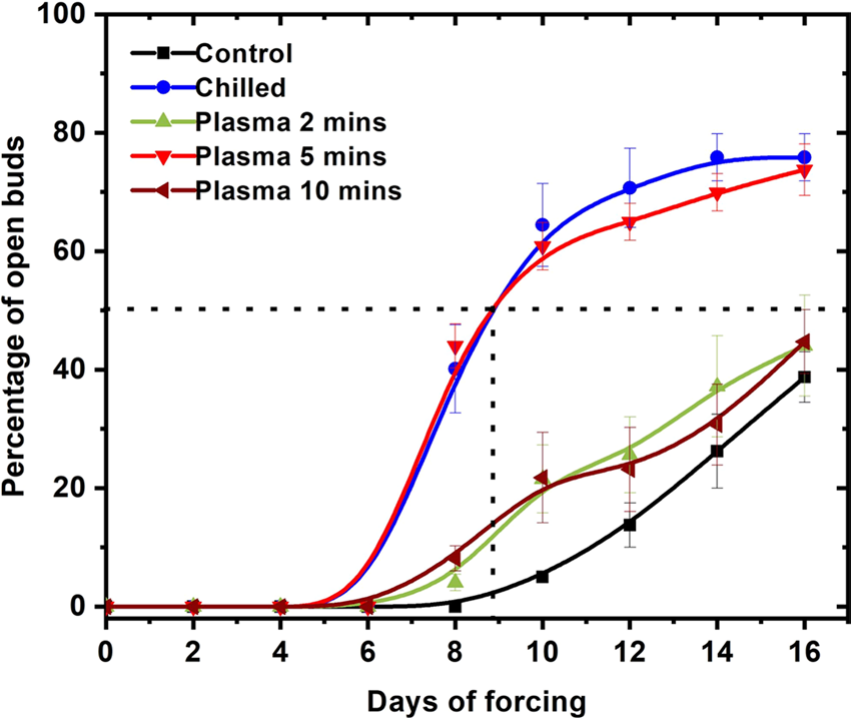
The percentage of buds which reached budburst stage as a function of days of forcing under 22 °C, 14 H light- and 18 °C, 10 H dark-period conditions on Muscat of Alexandria grape shoot cuttings. Prior to forcing, the cuttings were either untreated (control), chilled for 1-month at 4 °C or treated with cold atmospheric plasma for 2, 5 or 10 min. Each point represents the mean ± SE of four replicates of 20 cuttings each.

The percentage of buds which reached budburst stage as a function of days of forcing is shown in Fig. 3. In comparison to untreated shoot cuttings, much higher percentages of open buds were achieved by the 5-minutes plasma and 1-month chilling treatments (74% and 76%, respectively). The final percentage of budbreak was ~ 39% on untreated cuttings. The 2- and 10-min plasma-treated buds started opening slightly earlier than the control; however, the final percentage after 16 days of forcing was comparable to the control at ~ 44%.

**Figure 4:**
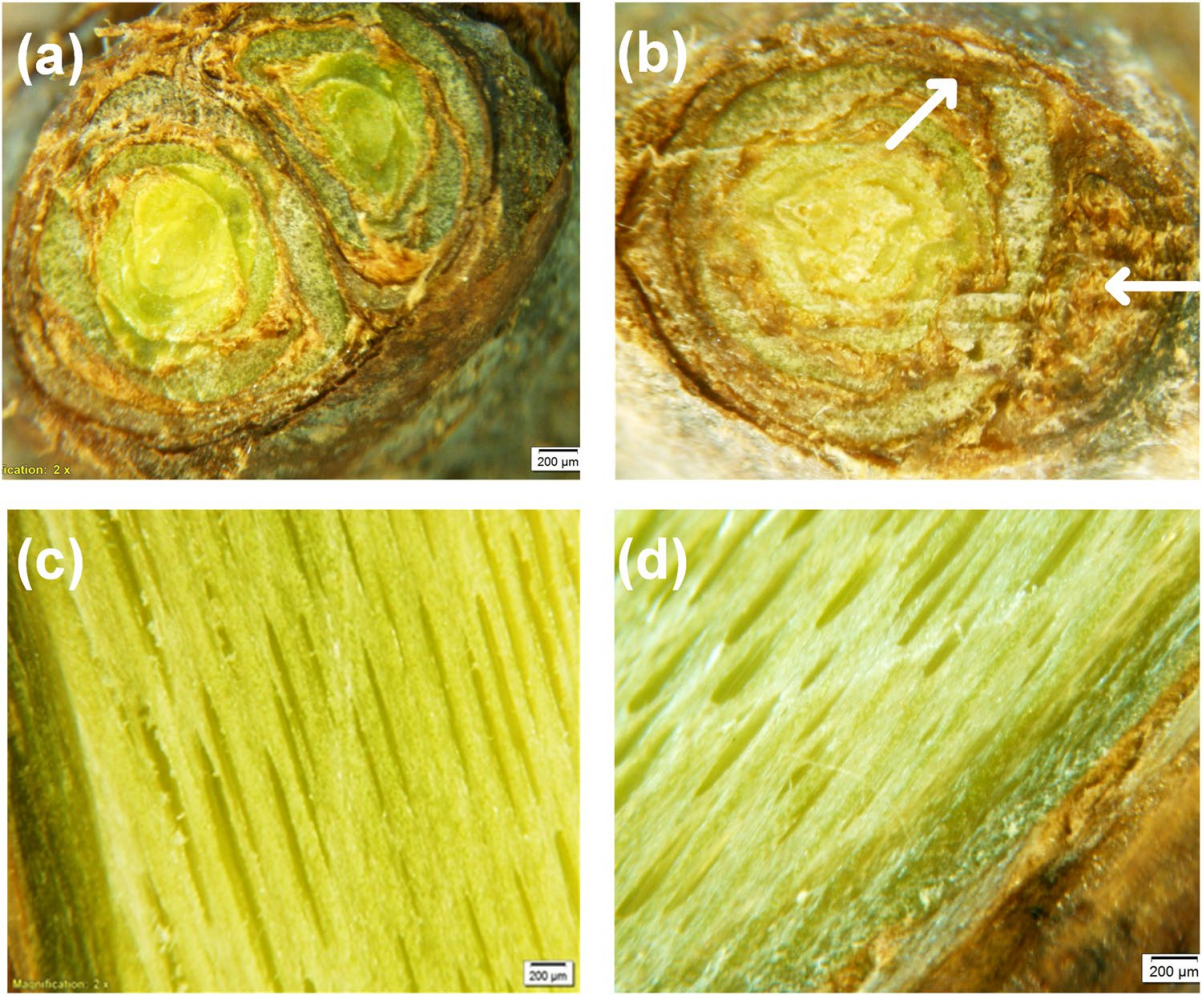
Above, cross-sections of dormant buds of Muscat of Alexandria grape after three days of forcing under 22 °C, 14 H light- and 18 °C, 10 H dark-period conditions. Prior to forcing, the cuttings were either untreated (control) (**a**) or treated with cold atmospheric plasma for 10 min (**b**). Below are photographs of vascular tissues from the stem below the bud of control (**c**) and plasma-treated cuttings (**d**). Arrows indicate brown tissue potentially damaged by exposure to plasma.

Close examination of cross-sections of several plasma-treated buds did not reveal any damage to the primary bud within the compound buds (Fig. 4b). However, we do see some darkening of some secondary buds and the peripheral tissues in the primary buds treated with plasma for 10 min possibly due to damage to these tissues. The vascular tissues underlying the bud do not show any signs of damage (Fig. 4c).

**Figure 5:**
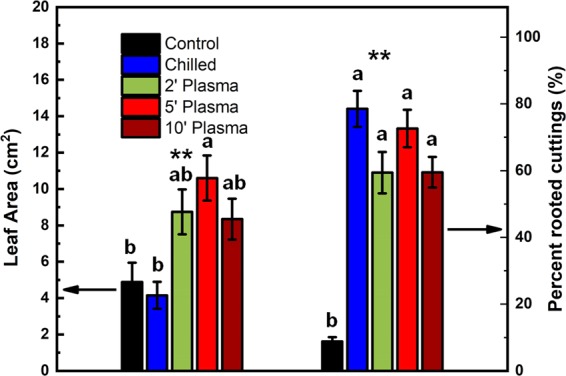
Average leaf area (left) and percentage of rooted cuttings (right) in untreated control, 2-, 5- and 10-min plasma treated and 1-month chilled Muscat of Alexandria grape shoot cuttings after 16 days of forcing under 22 °C, 14 H light- and 18 °C, 10 H dark-period conditions. Each bar represents the mean ± SE of four replicates of 20 cuttings each. For each tissue, the bars marked with different letters represent means which are significantly different at *P* < 0.05. ^**^The differences among the treatments are significant at *P* < 0.01.

The comparison of the average area per leaf of new shoots from each treatment after 16 days of forcing is shown in Fig. 5 (Left). This is the area of the largest leaf on each cutting which grew and then the average area per leaf from each replicate was calculated. Plasma-treated cuttings had larger leaves then control and chilled cuttings (*P* < 0.01). The leaf area increased from 4.9 cm^2^ in control buds to ~8.7, 10.6 and 8.3 cm^2^ after 2, 5 and 10 minutes of plasma treatment, respectively. The 5 min treatment gave a larger leaf area than the chilled and control (*P* < 0.01), whereas the 10 and 2 min treatments tended to have larger leaf area than the control and chilled treatments (*P* = 0.14 and 0.22, respectively). Surprisingly, under these criteria the leaf area of chilled buds was slightly smaller than that of control buds (4.1 cm^2^). Exposure to plasma or cold appears to have a profound effect on rhizogenesis (Fig. 5 – Right). The percentage of stems which developed roots sharply increased in all four treatments compared to control (*P < *0.01).

**Figure 6:**
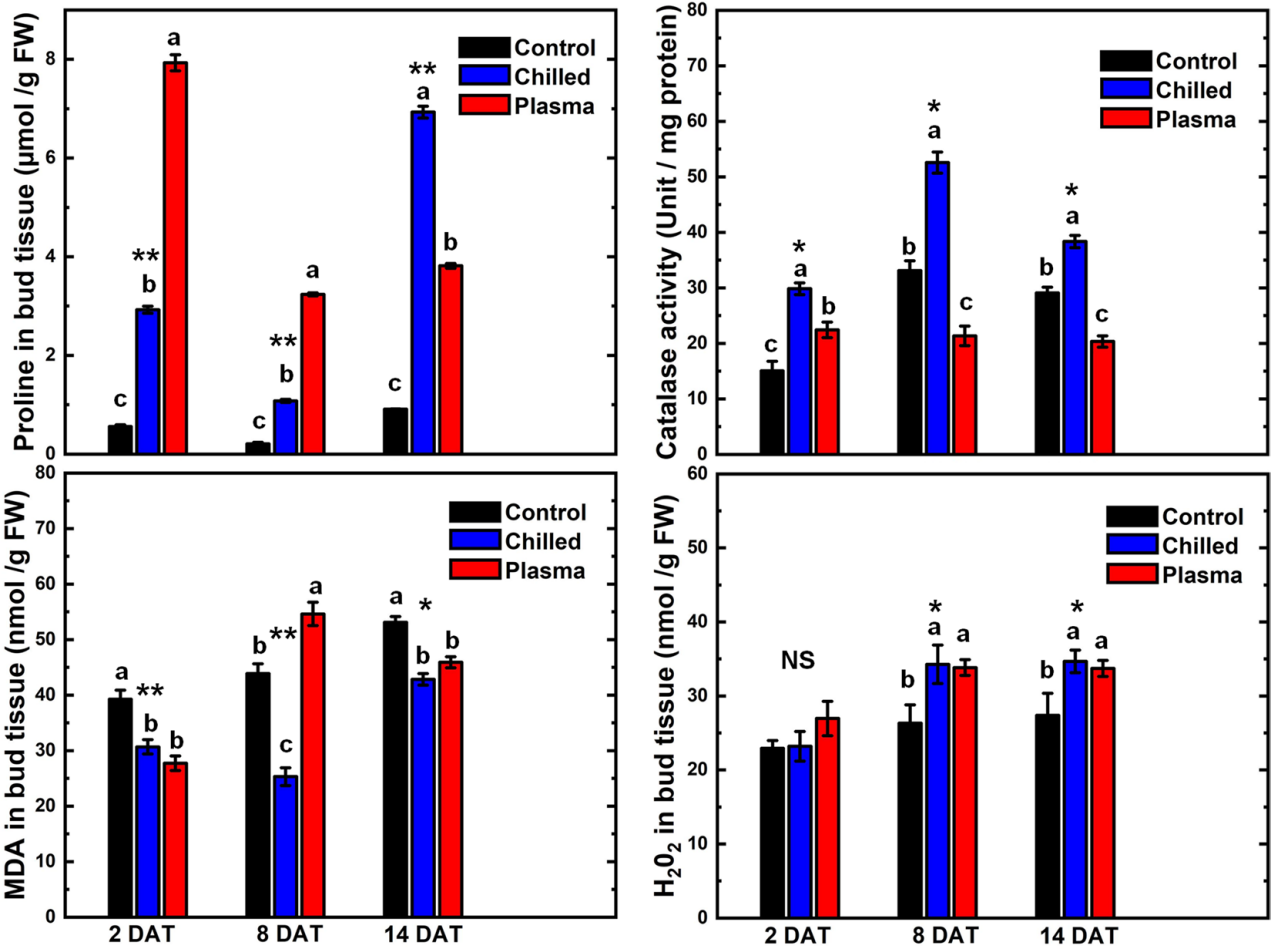
The concentrations of free proline, malondialdehyde (MDA) and hydrogen peroxide (H2O2) and catalase activity (CAT) in bud tissue of Muscat of Alexandria grape shoot cuttings after 2, 8 and 14 days of forcing under 22 °C, 14 H light- and 18 °C, 10 H dark-period conditions. Prior to forcing, the cuttings were either untreated (control), chilled for 1-month at 4 °C or treated with cold atmospheric plasma for 5 min. Each bar represents the mean ± SE of three determinations. For each date, the bars marked with different letters represent means which are significantly different at *P* < 0.05. ^NS, *, **^ For a given date, the differences among the treatments are not significant, or significant at *P* < 0.05 or <0.01, respectively.

Figure 6 shows the change in the concentrations of free proline, MDA and H_2_O_2_ and CAT activity in bud tissues of Muscat of Alexandria grape shoot cuttings after 2, 8 and 14 days of forcing under warm long day conditions. After two days of forcing, 5-min plasma-treated samples had the highest concentration of proline (*P* < 0.01) (Fig. 6). Compared to the control samples which had 0.56 µmol g^−1^ FW, the 5 min plasma-treated samples had a phenomenally high proline concentration of 7.85 µmol g^−1^ FW, i.e. almost fourteen times higher. The chilled buds had a concentration of 2.85 µmol g^−1^ FW, which is much higher than the control but about a third of what was measured in plasma-treated buds. On day 8 of forcing, proline concentrations were significantly less than on day 2 for all three treatments. However, still, the 5 min plasma-treated buds had the highest concentration at 3.3 µmol g^−1^ FW. While the control and the chilled buds had 0.2 and 1.05 µmol g^−1^ FW, respectively. On day 14, the concentration of proline in plasma-treated buds did not change significantly but it increased markedly in control and chilled buds.

After two days of forcing, the concentration of MDA was slightly higher in the tissues of untreated buds compared to cold- and plasma-treated buds (Fig. 6) (*P* < 0.01). Control buds had a MDA concentration of 37.7 nmol g^−1^FW of bud tissue. Six days later, the concentration increased in untreated and plasma-treated buds but not in chilled ones. MDA concentrations changed to 43.7, 25.7 and 57.1 for the control, chilled and 5 min plasma treatments, respectively.

The change with forcing time in CAT activity (unit mg^−1^ protein) in untreated, chilled and 5 min plasma treated cuttings is shown in Fig. 6. After two days of forcing, control buds had the lowest activity of CAT, with 15-unit mg^−1^ protein (*P* < 0.05). The activity was higher in cold- and plasma-treated buds. After 8 days of forcing, CAT activity almost doubled in control and chilled buds but remained unchanged in plasma-treated buds. Just like with proline, CAT activity increased after 8 days of forcing in chilled samples compared to control. The activity in the cuttings which received these two treatments decreased slightly on day 14 of forcing; whereas, in plasma-treated buds the level of CAT activity remained the same throughout the forcing period.

The change over time of H_2_O_2_ concentration in untreated, chilled and 5 min plasma treated shoot cuttings is shown in Fig. 6. After two days of forcing, plasma-treated buds tended to have a slightly higher concentration of H_2_O_2_ than those which received the other treatments (*P* = 0.12). On days 8 and 14 of forcing, chilled and plasma-treated buds had higher concentrations of H_2_O_2_ than untreated buds in which the concentration changed little over time (*P* < 0.05).

## Discussion

The results show that the final percentage of budbreak on cuttings which were exposed to plasma for 5 minutes or to chilling temperature for one month increased by ~100% over that of control cuttings. Also, cuttings treated with plasma for 5 min reached 50% budbreak 9 days ahead of untreated cuttings. The 2 and 10 min plasma-treated buds started opening slightly earlier than the control; however, the final percentage of budbreak was comparable to that of untreated cuttings. This indicates that the 2 min was probably not enough to stimulate budbreak, whereas the 10 min may have started damaging the outside layer of the buds. In fact, a close examination of cross-sections of several plasma-treated buds for 10 min did not reveal any damage to the primary buds within the compound buds; however, some secondary buds and the peripheral tissues in some primary buds turned brownish indicating possible damage to these tissues. Furthermore, a small percentage of the buds may have been fatally injured as suggested by the fact that about 25% of the buds appeared dormant after 16 days of forcing compared to less than 5% on untreated cuttings. The vascular tissues underlying the bud did not show any signs of damage. Therefore, the reduction in budbreak on cuttings exposed to plasma for 10 minutes may have been caused by an inactivation of some of their primary buds rather than an outright killing of the meristems. There is no previous work with plasma on buds to compare with, but experiments on seeds show that there is an optimum duration of exposure to plasma beyond which germination decreases^22^.

The comparison of sprouting vigour, as inferred from the size of the new leaves, indicates that exposure to plasma had a stimulating effect on shoot growth. The average area per leaf for each treatment was largest for the plasma-treated cuttings and especially the 5-minute treatment. In addition to releasing the dormancy of meristems and predisposing them for fast growth resumption, plasma may have also suppressed fungal growth thus allowing leaves to become larger. It is known that non-thermal plasma treatment reduces the fungal growth and up-regulates resistance genes in plants^40,41^.

A surprising finding was the positive effect of plasma on rhizogenesis. The final percentage of cuttings which developed roots was more than 70% for cold and 5 minutes plasma treatments compared to ~60% for the 2- and 10-minutes plasma and less than 10% for the control. This shows that even a short exposure to plasma affects the shoots in a way similar to chilling. Plasma treatment encouraged both budbreak and root differentiation as efficiently as cold temperature. The percentage is much higher in all treated samples compared to the control samples. This indicates that the plasma treatment altered the hormonal balance of the cuttings in favour of sprouting and rhizogenesis. Similarly, when tobacco seedlings were subjected to drought stress, they accumulate auxin and develop more lateral roots^42^.

So far, a direct relationship between the ROS signaling and changes in membranes functioning and structure has not been reported for dormant buds. However, it has been shown that the hydroxyl radical OH plays a direct role in processes leading to membrane loosening in germinating seeds^43^. It is therefore possible that ROS signaling could be involved in cell wall loosening leading to the release of bud dormancy. Numerous studies were carried out to unravel the mechanisms underlying the process of dormancy release in buds of temperate zone tree and vine species. Most of these studies agree that stresses, especially oxidative and respiratory stresses, play a role in the release of bud dormancy^13,15,44,45^. Due to these stresses, ROS are produced which affects the growth and development of plant in various ways^44^. For instance, exogenous application of H_2_O_2_ can substitute for chilling by initiating a series of events which eventually lead to the release of bud dormancy^46^. Still ROS in high levels have harmful effects and must be strictly controlled to fulfill their role as cellular messengers^44^. Hence, the antioxidant control systems involving the enzymes CAT, superoxide dismutase (SOD) and peroxidase (POD) are upregulated during dormancy release to check the level of ROS in plant tissues. In grape buds, the interconnections between bud dormancy status, CAT activity and H_2_O_2_ concentration levels were well demonstrated. It was found that the application of CAT inhibitors or exogenous H_2_O_2_ induce dormancy breakage by favoring the oxidative pentose phosphate pathway (PPP) which presumably provides reducing power and carbon for the new growth^47^.

In the present study, the concentration of H_2_O_2_ in cold-treated buds early in the forcing period was comparable to its level in control buds possibly due to the higher CAT activity; therefore, membrane lipid peroxidation, as indicated by MDA concentration, was lower than what was detected in control buds. As budbreak proceeded and growth accelerated, one would presume that nitrogen and sugar reserves were mobilized and respiration intensified with potentially more production of H_2_O_2_. In response, CAT activity increased but apparently not enough to prevent an increase in lipid peroxidation (Fig. 6).

CAT activity was slightly higher in plasma-treated tissues compared to control buds but remained unchanged throughout the forcing period as opposed to the activity in control and chilled buds which increased markedly compared to the start of forcing. Understandably, the level of H_2_O_2_ increased too as forcing proceeded leading to some membrane damage as indicated by the higher concentration of MDA and the browning of peripheral tissues in some buds. Our results show that CAT activity, the main H_2_O_2_ degrading enzyme, increased gradually in the chill-treated buds of grapevine during dormancy breakage, and this response was parallel to a slight increase in the level of H_2_O_2_ and a decrease in the level of MDA after 8 days of forcing (Fig. 6). This indicates that the strong activity of CAT in the chill-treated buds either at the beginning of the forcing period or after 8 days effectively guarded against peroxidation of cell membrane lipids. Nevertheless, the slight increase of H_2_O_2_ in chilled bud tissues may have generated a moderate oxidative stress or may have acted as a chemical signal that triggered the expression of genes related to endodormancy release^48,49^. In addition, chilling treatment led to a quick accumulation of free proline in the bud tissues (Fig. 6). This common stress response was previously reported in response to treatment with cold^50^ or hydrogen cyanimide^16,51^. Still the highest increase in proline content was caused by the 5-min plasma treatment (*P<0.01)*, which peaked after 2 days of forcing then decreased possible due to turnover and for being used as a source of nitrogen and carbon by the developing bud structures. The high level of proline was matched by a low CAT activity. Proline was shown to increase the activity of POD and decrease the activities of CAT and SOD in grapevine tissue^52^. A similar result was reported when grape buds were sprayed with hydrogen cyanimide^14–16^. Accordingly, compared to chilling, it appears that the plasma treatment triggered a more intense but transient oxidative stress as indicated by the temporary increase of MDA content especially on the 8^th^ day of forcing. Such oxidative stress is part of the mechanism leading to budbreak in both treatments. Furthermore, the sharp increase in proline biosynthesis in the case of plasma treatment may have led to an increase in NADP^+^/NADPH ratio and then the activation of the oxidative pentose phosphate pathway which is important for antioxidative defense and dormancy breakage^47,53^. Proline can also stabilize sub-cellular structures, quench active oxygen and protect cells against the adverse effects of stress^54^. The response of grape buds to cold plasma appears similar to that of chilling and artificial rest-breaking chemicals^48,55^. Proline seems to play a key role in this response: when it accumulates, it protects the cells against oxidative stress and upregulates the oxidative pentose phosphate pathway causing a series of events which lead to the release of dormancy. When the stress abates, proline is degraded giving way to new metabolite compounds used by anabolic pathways which yield the ATP, phosphorylated sugars and amino-acids needed to initiate budbreak and active growth resumption^51^.

## Conclusion

In this work, we report on a novel plasma-based environmentally friendly treatment of dormant grape buds. The plasma-based treatment has shown a large improvement of several growth parameters including an earlier and more synchronous bud growth and a higher final percentage of budbreak compared to untreated buds. Cuttings exposed to plasma for 5 minutes also had larger leaves and developed more roots.

Several previous studies suggested that the release of buds dormancy involves stresses, especially oxidative stresses. In grape buds, the interconnection between dormancy release and oxidative processes is well established. When the buds were exposed to plasma, their tissues accumulated the amino-acid proline and H_2_O_2_ whereas CAT activity remained stable; this appears to have led to an intense but transient oxidative stress which induced the meristems to resume active growth and the buds to open earlier and in larger numbers. It appears that H_2_O_2_ and proline are key factors in the process; the former directly or indirectly caused the oxidative stress while the latter helped protect cell membranes against free radicals and possibly upregulated the oxidative pentose phosphate pathway prompting a chain of events leading to dormancy release.

We anticipate that our work will provide a starting point for the development of novel plasma-based tools and methods to treat dormant plants whether *ex-situ* or in the field. This technique will be an effective and safer alternative to chemical dormancy-breaking substances. Furthermore, the plasma treatment method may allow higher agricultural production in several regions of the world, at risk of becoming marginal due to global warming^7^.

## Supplementary information

Supplementary information

## Data Availability

The datasets generated during and/or analysed during the current study are available from the corresponding author on reasonable request.
