# Supplementary material for: Cold plasma treatment to release dormancy and improve growth in grape buds: a promising alternative to natural chilling and rest breaking chemicals
Source: Sci Rep. 2020 Feb 14;10:2667. doi: 10.1038/s41598-020-59097-x (PMC7021807; doi:10.1038/s41598-020-59097-x)
Supplement: Supplementary file 1 — Supplementary information [file 41598_2020_59097_MOESM1_ESM.docx]

**Cold plasma treatment to release dormancy and improve growth in grape buds: a promising alternative to natural chilling and rest breaking chemicals**

Z. Mujahid, T. Tounekti, and H. Khemira


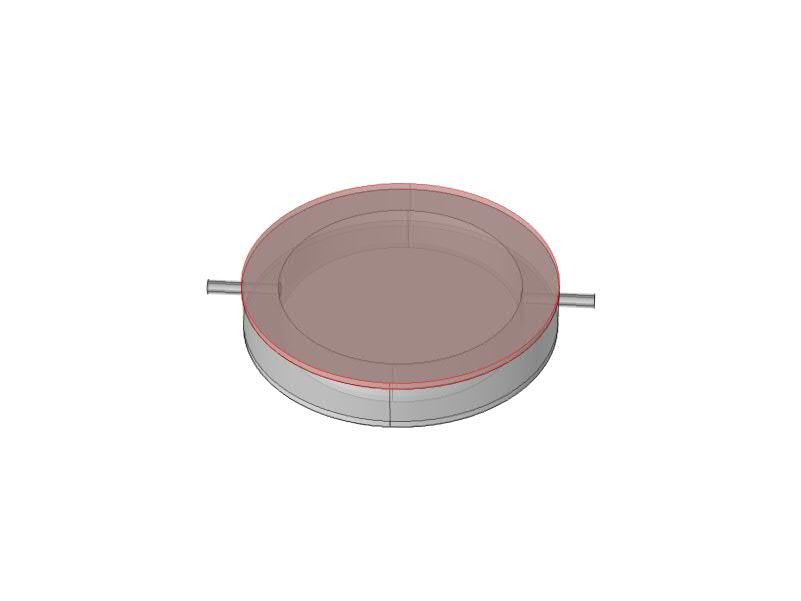


Supplementary Figure S1 shows the schematic of a parallel plate dielectric barrier discharge used for the plasma treatment in this work. The DBD cell consists of a 1 cm thick quartz ring with two pipe connectors for gas inlet and outlet. The top and bottom plates are made of glass and the top plate can be opened to insert the bud samples. The electrodes are attached to the outer sides of the glass plates. The plasma is generated with a 10 kHz sinusoidal power supply at a fixed power of 30 W.
